# Registry-Based Pragmatic Trials in Heart Failure: Current Experience and Future Directions

**DOI:** 10.1007/s11897-017-0325-0

**Published:** 2017-02-28

**Authors:** Lars H. Lund, Jonas Oldgren, Stefan James

**Affiliations:** 1 Department of Medicine, Unit of Cardiology, Karolinska Institutet, Solna, Sweden; 2 Department of Cardiology, Karolinska University Hospital, 117 76 Stockholm, Sweden; 3 Uppsala Clinical Research Center and Department of Medical Sciences, Cardiology, Uppsala University, Uppsala, Sweden

**Keywords:** Heart failure, Registry, Prospective randomized clinical trial, Pragmatic clinical trial, Registry-based pragmatic trial, Registry-based prospective randomized clinical trial, Cost

## Abstract

**Purpose of Review:**

Randomized controlled trials (RCTs) in heart failure (HF) are becoming increasingly complex and expensive to conduct and if positive deliver expensive therapy tested only in selected populations.

**Recent Findings:**

Electronic health records and clinical cardiovascular quality registries are providing opportunities for pragmatic and registry-based prospective randomized clinical trials (RRCTs). Simplified regulatory, ethics, and consent procedures; recruitment integrated into real-world care; and simplified or automated baseline and outcome collection allow assessment of study power and feasibility, fast and efficient recruitment, delivery of generalizable findings at low cost, and potentially evidence-based and novel use of generic drugs with low costs to society.

**Summary:**

There have been no RRCTs in HF to date. Major challenges include generating funding, international collaboration, and the monitoring of safety and adherence for chronic HF treatments. Here, we use the Spironolactone Initiation Registry Randomized Interventional Trial in Heart Failure with Preserved Ejection Fraction (SPIRRIT-HFpEF), to be conducted in the Swedish Heart Failure Registry, to exemplify the advantages and challenges of HF RRCTs.

## Introduction

### Challenges in Heart Failure

Heart failure (HF) affects 2% of the population and up to 20% of the elderly [1], is the most common cause of hospitalization [2•], and is associated with mortality of approximately 20% at 1 year [3]. The prevalence will increase with an aging population, and direct costs for HF are expected to increase threefold between 2010 and 2030 [4].

In HF with reduced ejection fraction (HFrEF), a generation of trials of drug and device therapy has substantially improved prognosis [5, 6]. A major challenge in this phenotype has become proper utilization of existing interventions and improved implementation in under-served patient groups [7–11] as well as testing efficacy in previously excluded populations such as those with chronic kidney disease or hyperkalemia [12].

In contrast, HF with preserved EF (HFpEF) affects about half of the HF population [5, 13, 14•, 15], is increasing in prevalence, and will be the dominant form in the future [16]. Neurohormonal antagonist drugs that are now generic and inexpensive appear promising [3, 17–19], but have not convincingly been demonstrated to be beneficial in trials [20–23], and evidence-based therapy for HFpEF has been identified as “the greatest unmet need in cardiovascular medicine” [24••]. Furthermore, positive outcome trials have generally included patients with EF ≤40%, whereas EF would be considered preserved or normal only if ≥50% [25], leaving a mid-range category recently recognized as in particular need of further study [5, 14•] and where neurohormonal antagonist drugs may be reasonably expected to have potential benefit [26, 27].

Acute HF (AHF) is considered a distinct phenotype [28–30]. Like in chronic HFpEF, prognosis has not improved over time. Over 50% of patients are discharged with unresolved symptoms, and within 60 days, 50% have again worsening symptoms, 25% are re-hospitalized, and over 10% have died [1, 31]. As in HFpEF, trials of novel interventions have been largely unsuccessful. There are multiple supportive generic and inexpensive treatments with class IIa–IIb recommendations in guidelines but no underlying evidence [5, 6, 30].

## The Complexity of the Randomized Controlled Trial

A foundation for contemporary clinical decision-making is the evidence from prospective randomized clinical trials (RCTs), which have transformed medical practice over the last 70 years [32•]. When patients are randomized to intervention and control groups, bias and confounding are eliminated, and causality between the intervention and the outcome is established. Indeed, the establishment of RCTs and evidence-based medicine has been ranked among the most important medical discoveries of any kind, together with, e.g., vaccines, antibiotics, and the discovery of DNA [33].

However, while the concept of randomization is simple, RCTs have become increasingly large, complex, and expensive, which may threaten their very existence [34]. Design and reporting of RCTs is sub-optimal. Of 96,346 studies registered in ClinicalTrials.gov between 2007 and 2010, a majority were small and with heterogeneous reporting of methodology [35]. Among 13,327 trials between 2008 and 2013, only 13% reported results within 12 months of completion [36]. Among 244 extramural trials funded by the National Heart, Lung, and Blood Institute (NHLBI) and completed between 2000 and 2011, only 64% had been published by 2012 and median time to publication was 25 months [37]. Although RCTs in cardiovascular (CV) medicine have transformed CV care, in a review of 16 disease-specific, diagnostic, and interventional CV guidelines, only 12% of recommendations were level A (with HF the highest at 26% and valvular disease the lowest at 0.3%), and 48% of recommendations were level C [38].

## Registry Studies Serve Many Functions but Are Observational

**Fig. 1:**
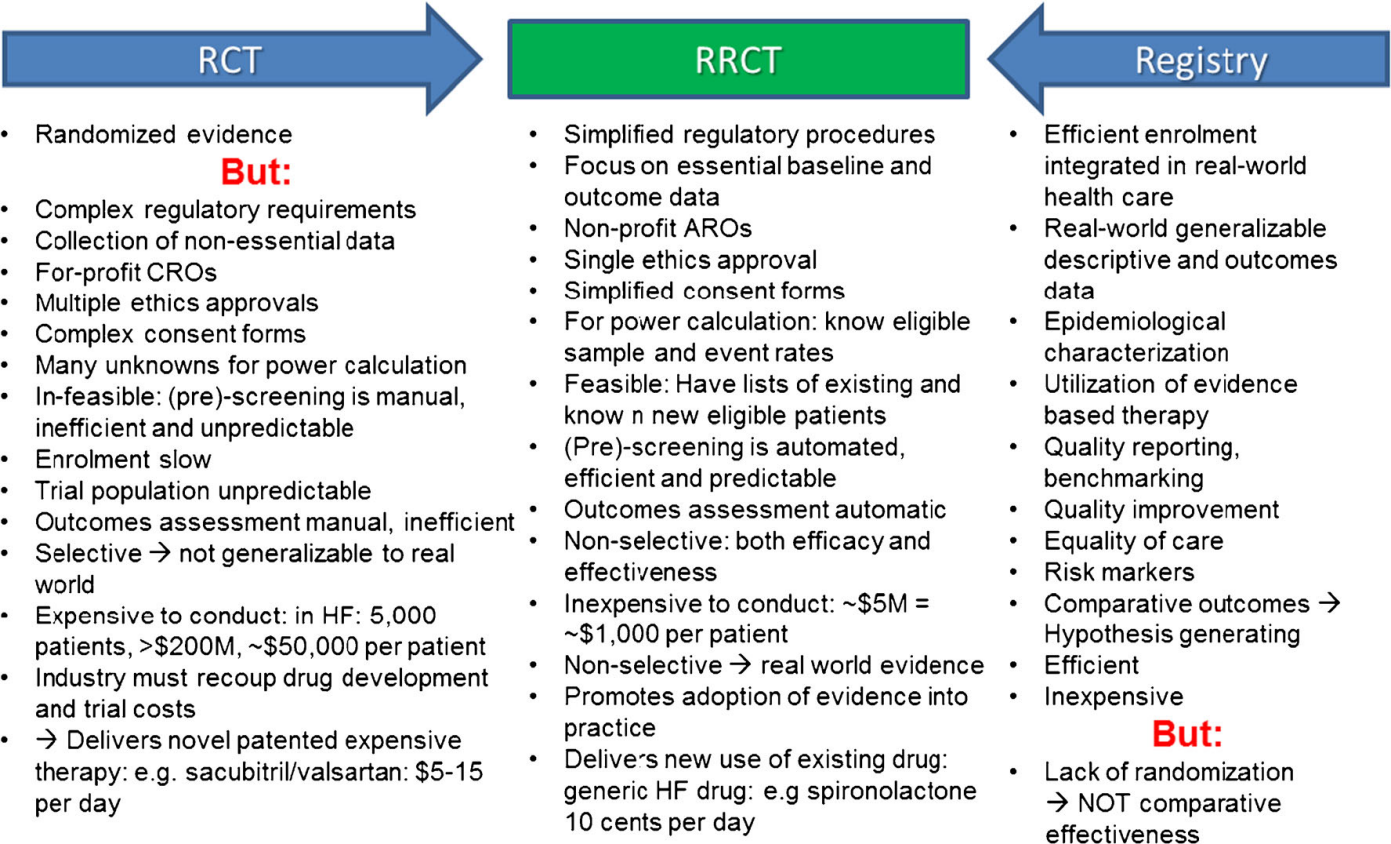
Characteristics or RCTs and registries, and advantages of RRCTs. Each item is discussed in text. *RCT* prospective randomized controlled trial, *RRCT* registry-based prospective randomized controlled trial, *CRO* contract research organization, *HF* heart failure, *M* million, *ARO* academic research organization

CV disease is common and associated with significant event rates, and there is a plethora of available interventions. This lends itself to and indeed demands systematic quality reporting, and registries in cardiovascular medicine have evolved over the last 20–30 years [39–41, 42•]. Registries serve many functions including quality reporting and improvement; benchmarking and performance measures; assessment of practice patterns and trends, outliers, and safety signals; and patient empowerment and standardization and promotion of equality of and access to care. They provide extensive generalizable (externally valid) data at low cost and high efficiency [39–41, 42•, 43•, 44] (Fig. 1) and are suitable for studying clinical associations, risk markers, potential risk factors, and risk scores [44–46]. HF registries including the Swedish Heart Failure Registry (SwedeHF) [7–9, 47–62] have characterized use of evidence-based interventions in different regions and contributed to improved utilization, and recently, we showed that enrolment in a HF quality registry, SwedeHF, was associated with lower mortality specifically explained by improved use of evidence-based CV and HF interventions [43•].

Clinical registries are part of real-world routine care and may therefore in comparison to clinical trial databases be limited by missing data, lower data quality, and lack of adjudication. Data not missing at random (NMAR) introduce bias and confounding but can be addressed by multiple imputation. Due to the complexity of the data, statistical methods are often more complex and may produce a “black box” impression and be intimidating for the clinical reader. However, in contrast to trial databases, registries are representative of most patients, which increases generalizability and external validity.

However, the most important limitation of observational studies is the lack of randomization of interventions, and thus the inability to determine treatment efficacy [40, 63]. It has been argued that the magnitude of associations in observational studies generally are similar to the magnitude of efficacy in RCTs [64, 65], but the lack of randomization inevitably produces bias and confounding [40, 63]. In a study of the associations between renin-angiotensin-system antagonist use and mortality in SwedeHF, the hazard ratio for death in HFrEF was 0.80 (95% CI 0.74–0.86; *p* < 0.001) [18], which precisely matches that in a large meta-analysis of RCTs in HFrEF [66]. The propensity score-matched hazard ratio in HFpEF was 0.91 (0.85–0.98; *p* = 0.008). However, although this closely matches the nominal hazard ratios for the primary outcomes in CHARM-Preserved (0.89; 0.77–1.03; *p* = 0.118) [20], PEP-CHF (0.92; 0.70–1.21; *p* = 0.545) [21], and TOPCAT (0.89; 0.77–1.04; *p* = 0.14) [23], these trials did not reach statistical significance, and renin-angiotensin-aldosterone system inhibition is not recommended specifically for HFpEF [5, 6].

## Pragmatic Clinical Trials

The evaluative or pragmatic clinical trial (PCT) was originally conceived to answer questions faced by decision makers and was distinguished from mechanistic or explanatory trials [67]. However, even conventional phase 3 RCTs are primarily evaluative. There are mechanistically sound phase 2 HF trials with positive surrogate endpoints that have failed in phase 3. Conversely, interventions such as sacubitril/valsartan [68] were effective in phase 3 in the absence of preceding phase 2 trials and with little understanding of mechanisms responsible for the clinical benefit.

PCTs are now more broadly considered those with large sample sizes; representative populations and generalizable and relevant outcomes; efficient use of existing resources; simplified operations (limited monitoring, safety reporting, trial-specific assessments, and regulatory and compliance documentation); baseline and if possible outcome data collection embedded in routine care setting or using telephone or automated follow-up; leveraging of electronic health records (EHRs) and registries; and simplified case report and informed consent forms [39–41, 69•, 70, 71]. The pragmatic features in trials exist inevitably on a spectrum, and many trials may be considered hybrid. The Pragmatic–Explanatory Continuum Indicator Summary (PRECIS) tools has been developed for characterization of pragmatic trials along this spectrum [72].

The literature describes numerous PCTs for diverse conditions and interventions [73•, 74]. In the CV field, the GISSI investigators’ trial of thrombolytics in myocardial infarction [75] and the International Studies of Infarct Survival (ISIS) [76] were early pragmatic trials, as were ALLHAT [77] and SAFE-PCI [78]. The ongoing ADAPTABLE (Aspirin Dosing: A Patient-centric Trial Assessing the Benefits and Long-term Effectiveness) trial from the Patient-Centered Outcomes Research Institute’s (PCORI) National Patient-Centered Clinical Research Network [79•] is a chronic intervention PCT enrolling 20,000 patients and is notable for efficient leveraging of electronic health records data.

However, with the exception of SAFE-PCI, these PCTs have not utilized registries, which have the added benefit of baseline and in some case outcome data being embedded in routine clinical care or accessible by automatic linking of data sources. Existing registries in heterogeneous health systems collect baseline data but often do not have access to outcomes. In HF, the US Get With The Guidelines (GWTG)-HF registry has been associated with improved utilization of HF interventions [54], but we are not aware of efficient and reliable ways of linking outcomes to this registry. There have been some smaller pragmatic HF trials in disease management and self-care [80–82] but none with drug or device interventions. The NHLBI-sponsored Heart Failure Network has conducted several trials at comparatively low cost but has to our knowledge not incorporated specific pragmatic features. ASCEND-HF was managed by a consortium of academic research organizations (AROs) rather than contract research organizations (CROs), but most other aspects of the trial were conventional, and the intervention, nesiritide, was not chronic [83].

## Limitations of RCTS and How They Can Be Mitigated in RRCTS: Future Directions

### Swedish Registries

The Swedish universal standardized publicly funded health care system [84] together with unique personal identification numbers [85] is uniquely suited for an extensive registry infrastructure. Sweden has currently 96 quality registries funded by the federal and regional governments, coordinated by the Swedish Association of Local Authorities and Regions (www.skl.se) and described at www.kvalitetsregister.se. Among cardiovascular registries are the Swedish Web-system for Enhancement and Development of Evidence-based care in Heart disease Evaluated According to Recommended Therapies (SWEDEHEART, www.ucr.uu.se/swedeheart) which includes myocardial infarction, percutaneous coronary and valve interventions, cardiac surgery, secondary prevention, and cardiogenetic disorders, and the Swedish Heart Failure Registry (SwedeHF, www.SwedeHF.se).

SwedeHF was founded in 2000 and is an ongoing syndrome-specific nationwide voluntary quality reporting registry with close to 110,000 registrations in 70,000 unique patients since 2000. The inclusion criterion is physician-judged HF. EF is recorded as <30, 30–39, 40–49, and ≥50%. Patients are enrolled at discharge from hospital or at out-patient encounters. Patients are informed of registration and opt-out is possible, but there is no written informed consent. Approximately 100 baseline clinical and medication variables are recorded in an online case report form (CRF) managed by Uppsala Clinical Research Center (UCR, www.ucr.se).

Sweden also provides governmental registries on health, medications, demographics, and national statistics. The Swedish Board of Health and Welfare (www.socialstyrelsen.se) maintains the mandatory National Patient Registry (NPR), Cause of Death Registry, and Dispensed Drug Registry. NPR collects International Classification of Diseases (ICD) diagnostic and procedure codes since 1987. The positive predictive values for most diagnoses are 85–95% [85]; a heart failure diagnosis was verified in 86–91% of cases [86]. NPR can be linked to the quality registries to provide additional baseline comorbidity data as well as cause-specific hospitalization and morbidity outcomes. The cause of death registry is based on death certificates and records both mode and underlying causes of death. It is not validated against medical records but each death certificate is manually reviewed and inconsistencies corrected. The Dispensed Drug Registry is maintained by the Swedish Board of Health and Welfare and contains details on all prescriptions actually dispensed by a pharmacy since 1 July 2005. All pharmacies are required to participate by law and coverage is 100%.

Thus, both baseline and outcome data can be obtained by linking quality registries to these administrative databases. Using this infrastructure, Sweden with addition of Iceland and one Danish site conducted the landmark RRCT Thrombus Aspiration in ST-Elevation Myocardial Infarction in Scandinavia (TASTE) trial, described in detail elsewhere [40, 87, 88]. There are additional ongoing trials in acute coronary syndromes, including DETOX-AMI (oxygen) and VALIDATE (bivalirudin vs. heparin) [40]. However, these are all testing acute interventions and do not require any follow-up or monitoring of chronic care. There have been no RRCTS to date in HF.

**Fig. 2:**
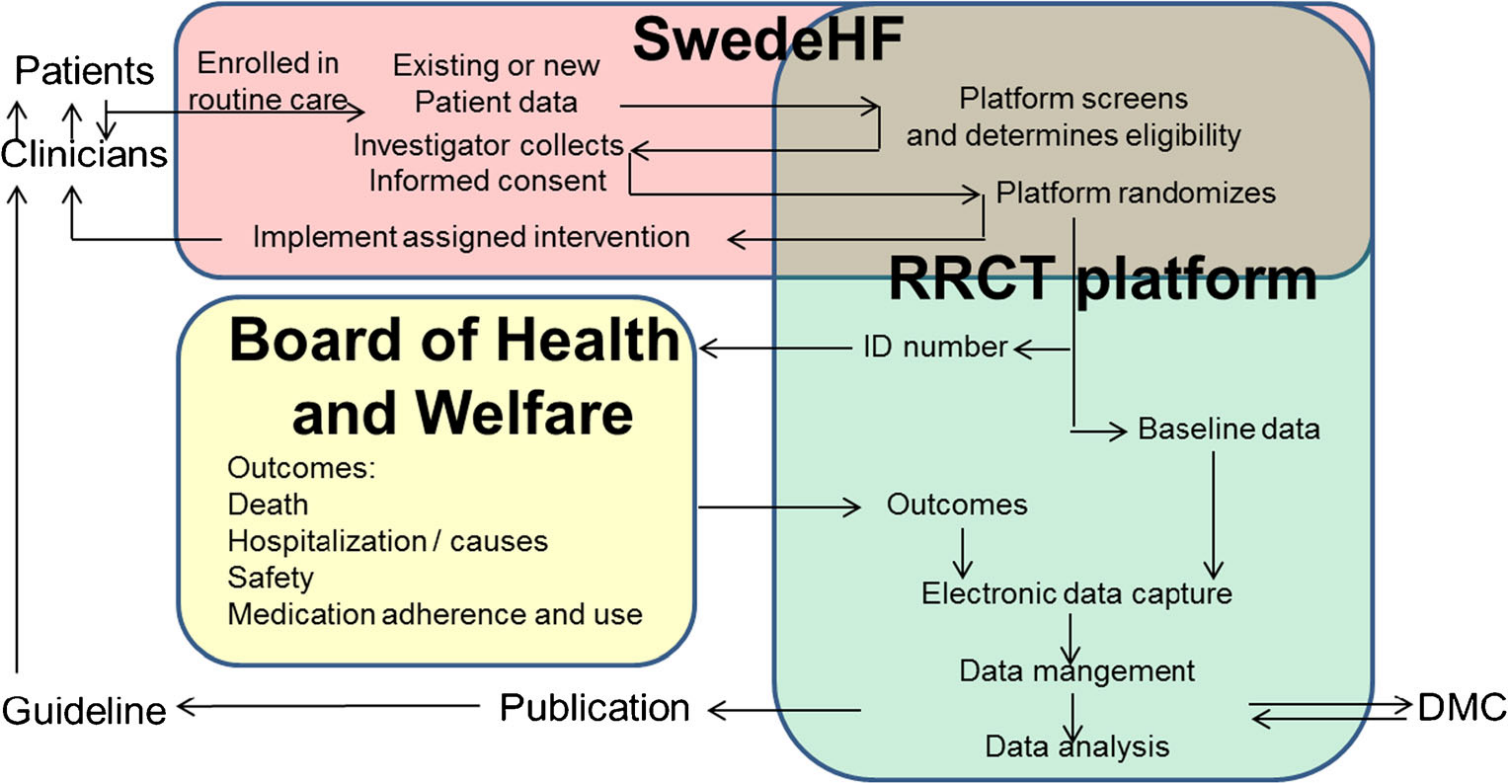
The integrated platform for RRCTs in the Swedish Heart Failure Registry. New patients are entered into the registry, and the platform screens and determines eligibility for both [1] previously entered and living patients, and [2•] online as new patients are entered. Eligibility is fed back to the investigator in the registry, in the form of [1] lists of existing eligible patients and [2•] online as entered patients are determined to be eligible. The investigator collects informed consent, the platform randomizes online and the investigator implements the randomized assignment and follows the patient in routine clinical care (with or without extra trial-specific follow-up). The baseline data entered into the registry as part of routine care together with randomized assignment are fed into a separate electronic data capture (EDC) system in the RRCT platform. The unique personal ID number is fed to the independent registries in the Swedish Board of Health and Welfare, which feeds back outcomes (deaths and causes, hospitalization and causes, new diagnoses, and medication use and randomized medication assignment adherence) to the EDC in the platform. Baseline and outcome data are then analyzed, provided to an independent data monitoring committee (DMC), and published. *SwedeHF* The Swedish Heart Failure Registry, *RRCT* registry-based randomized controlled trial

The Spironolactone Initiation Registry Randomized Interventional Trial in Heart Failure with Preserved Ejection Fraction (SPIRRIT-HFpEF) is an RRCT under development in SwedeHF. It is prospective randomized, multicenter, safety/efficacy, parallel assignment, intention-to-treat, open-label treatment, phase 4, event-driven interventional trial in HFpEF, testing spironolactone + usual care vs. usual care alone in patients with HF and EF ≥40%. To our knowledge, this is the first registry-based trial in heart failure. In the following, we present our view of the limitations of RCTs and the potential of RRCTs, and SPIRRIT-HFpEF will be used as an illustration and each section is summarized in Fig. 1. The RRCT platform and data linking for RRCT in SwedeHF is illustrated in Fig. 2.

### Regulatory Requirements and Non-essential Data

While necessary and justified from a good clinical practice (GCP) perspective, regulatory requirements are also becoming increasingly complex and both hindering trials [89••, 90, 91••, 92•] and indeed stimulating the growth of an entire for-profit contract research organization (CRO) industry. Sponsors and investigators often include extensive sub-studies and data collection, study visits, and questionnaires with little relevance to the main objectives but adding to cost and complexity [93•]. In CV phase III trials, there was a 50% increase in procedures and 30% increase in work burden between 2000–2003 and 2004–2007 [89••]. About 25% of trial procedures support regulatory requirements and noncore data [93•]. The typical trial in 2012 involved 13 endpoints, 169 case report form pages, and 175 days of on-site monitoring [94••]. The IMPROVE-IT trial [95] enrolled 18,144 patients and entailed 300,000 patient visits, 2.7 million CRF forms completed, over 15,000 SAEs processed, 14,709 events sent for adjudication, over 30,000 monitoring visits, 33 investigator meetings, and 9 DMC reviews (Dr. Michael Blazing, Duke University, personal communication).

Thus, a central feature of the RRCT is to reduce this complexity. SPIRRIT-HFpEF is sponsored by and will be managed by the non-profit academic research organization (ARO) UCR. Spironolactone is an approved and familiar drug, and in this phase 4 trial, there will be limited reporting of SAEs. Site monitoring will be risk-based and focus on consent procedures and inclusion/exclusion criteria. Randomization integrity is ensured by a central online randomization module, safety is monitored as an outcome, and the primary and all other endpoints are obtained automatically from national registries.

### Ethics and Informed Consent

The ethics and institutional review board review and approval in conventional RCTs are generally site-specific, complex, expensive, and time-consuming (Fig. 1). Single central ethics approval is more efficient and not inconsistent with GCP [69•, 70, 71]. Sweden has a national centralized ethics committee organization (www.epn.se) with local committees, each of which can provide a single centralized approval for multicenter studies (Fig. 1).

Written informed consent is a central tenet of GCP but also by definition excludes a proportion of otherwise eligible patients, slows recruitment and introduces selection bias, and limits generalizability [73•]. In large PCTs, it may be impractical with conventional consent procedures, and careful risk determination and the nature of the interventions may in some case allow waived or modified informed consent [73•, 96•, 97]. Individual consent may not be required for interventions that focus on clinicians or health systems, such as cluster-randomized trials [73•, 96•, 97], e.g., the Post-Myocardial Infarction Free Rx Event and Economic Evaluation, MI FREEE [98], or for e.g., patients with impaired consciousness, e.g., the Corticosteroid Randomization after Significant Head Injury trial, CRASH [99]. An integrated consent procedure has been proposed where consent is verbal and provided in the context of other patient decisions in usual care [97]. In TASTE, oral consent was collected prior to angiography, and written confirmation obtained after the randomized procedure. SPIRRIT-HFpEF has a single intervention, follow-up only by telephone (and automatically from registries) and no sub-studies, and a short and simple written consent form.

### Study Population, Screening, and Enrolment

In conventional RCTs, pre-screening is manual and often opportunistic (i.e., occurs as potential patients are encountered) and therefore inefficient and unpredictable (Fig. 1). Participation in trials has fallen over time, and the patient response rate to a screening invitation is generally less than 10% [73•] and likely lower in heart failure, where patients are often older, comorbid, and frail. Large trials generally include many hundred sites, but only 10% of clinicians participate in trials [100] and recruitment in CV trials is generally considerably less than 1 [101••] and in HF less than 0.4 patients per site per month, yielding a total of less than 20 per site [100]. This is a particular problem in the USA and Western Europe, whose share in multinational trial enrolment is declining [32•, 67, 73•, 102]. Embedding trial recruitment, randomization, and outcomes ascertainment into routine clinical care would improve enrolment and generalizability of trial findings [34, 71]. The EHR and in particular registries enable automated, online, and real-time screening (Fig. 1).

SwedeHF is widely accepted as an integral part of routine care, and there is broad participation throughout Sweden. This ensures both that the SPIRRIT-HFpEF trial will be supported and that the findings will be generalizable to Northern European patients with HFpEF. Patients will be automatically screened and recruited at enrolment in the registry (discharge from hospital or out-patient visit) and from a pool of living eligible patients, provided to each center by UCR (Figs. 1 and 2). Thus, early enrolment can be rapid from these existing patients. Known event rates in eligible patients in the registry facilitate the power calculation; known existing eligible patients and historical enrolment rates facilitate assessment of feasibility of recruitment. The only unknown is the proportion consenting. In TASTE, 60% of all STEMI patients and 77% of all eligible patients were included [88].

Many HFpEF and AHF trials may have failed in part because of suboptimal eligibility criteria or outcomes. The RRCT concept will not solve this problem but the existing registry can perform simulations that provide extensive data on the types of patients and events to expect given different eligibility criteria (Fig. 1). This use of registries is beginning to be recognized by and appealing to industry, whereas actual industry-sponsored trial conduct has not yet occurred in the registries.

### Intervention: Novel Treatment vs. Novel Use of Existing Treatment

Conventional RCTs are complex in part because they test *novel interventions*, proprietary unapproved drugs, or devices. Outcome adjudication and safety reporting become paramount. However, for HFpEF, there are numerous existing, approved, and generic interventions that may be tested in a *novel use* concept (Fig. 1). Spironolactone is approved for heart failure (without specifying EF). TOPCAT was promising but does not convincingly support its use, and there is consensus that a new trial such as SPIRRIT-HFpEF is needed [103]. Spironolactone will be prescribed by investigators and filled by patients at conventional pharmacies. Drug costs in Sweden are paid by the patient out of pocket up to an annual maximum of 2200 SEK (∼$240). Most patients will be at the max at all times of filling and re-filling study drug prescriptions. In rare occasions when study drug will incur personal costs, the local sites and in turn the sponsor will reimburse.

There are other interventions such as renin-angiotensin system (RAS) antagonists and diuretic regimens that are suitable for testing in HFpEF RRCTs. Many empiric interventions in AHF such as oxygen, diuretic regimens, and vasodilators are also inexpensive, familiar, and suitable for AHF RRCTs. Similarly, SwedeHF is suitable for prospective randomized or cluster-randomized trials of structured care, such as disease management, biomarker-driven, or self-care.

### Intervention: Acute vs. Chronic

TASTE and other PCTs tested acute intervention, with potential effects in the short or long term. No trial-specific follow-up may be needed if outcomes can be reliably obtained from registries. For chronic drug treatments, such as spironolactone in HFpEF, follow-up is needed both for adherence and safety. Adherence to spironolactone (and control) assignment will be monitored and enforced at four trial-specific follow-up telephone calls, at all usual care encounters, and in the Dispensed Drug Registry. Crossover will be defined for the control group as de novo spironolactone or eplerenone use. Prescriptions in Sweden are for 3-month durations but patients may have been intermittently adherent or taken reduced doses. Therefore, beyond information from direct patient contact, crossover will be defined in a treated patient as failure to refill within 6 months from baseline or from last dispensation, where crossover will be set to 3 months from randomization or last refill.

### Control, Placebo, and Blinding

In a double-blind RCT, randomization eliminates selection bias, and confounding and blinding eliminates treatment (placebo) and diagnostic bias. However, placebo entails considerable cost for procurement, packaging, labeling, transport, and storage, and accountability, and more importantly, increases the work-load on investigators and impairs recruitment. There are numerous examples of randomized but non-blinded trials that have met regulatory requirements and changed guidelines and standard of care, such as the RE-LY trial of dabigatran for atrial fibrillation [104], and trials of left ventricular assist devices in advanced HF [105, 106]. Pragmatic trials often do not include blinding and in order to minimize bias, focus on objective outcomes such as all-cause or CV mortality [73•] and/or use blinded endpoints in the prospective randomized open-label blinded endpoint (PROBE) design.

### Outcomes

Conventional RCTs often entail multiple endpoints, dependent on extensive event adjudication. Because interventions and drugs are novel, safety and SAE and AE reporting are extensive, and even trial endpoints are often concurrently reported as SAEs [94••]. While an independent clinical endpoints committee adjudicating non-fatal events is essential, the inclusion of numerous non-primary and complex endpoints often add unduly to the data collection and reporting requirements and burden to investigators.

Although many EHRs and registries may be suitable for patient identification, recruitment, and automated collection of baseline variables, a major limitation is the lack of efficient, reliable, and complete collection of outcomes. In addition to the large network of clinical quality registries in Sweden, the mandatory and complete coverage government health and statistical registries enable collection of outcomes (Fig. 1). In SPIRRIT, CV, HF, and all-cause hospitalization will be automatically collected from NPR, and CV and all-cause death from the cause of death registry (Fig. 2). Safety outcomes in aggregate will be reported from NPR. However, SPIRRIT-HFpEF will have some components of a conventional trial, specifically the monitoring of safety by measuring creatinine and potassium and the adjudication of CV death.

### Efficacy vs. Effectiveness

Conventional clinical trials are generally mechanistic and explanatory and optimized to determine efficacy [73•]. Already in 1967, concerns were raised that this design did not adequately inform clinical practice, with calls for pragmatic and evaluative trials [107]. Conventional RCTs systematically and non-randomly exclude certain patient groups such as the elderly and women [108, 109]. Indeed, the median age in many HF trials has been less than 70 years, as compared to 77 in HFpEF, 74 in HFmrEF, and 72 in HFrEF in SwedeHF. In PEP-CHF [21] and I-PRESERVE [22], the median NT-proBNP ranged 320–453 ng/L, whereas in observational studies of RAS-antagonists and beta blockers in SwedeHF, it was over 2000 ng/L [3, 18]. A conventional trial with extensive follow-up and time commitment on part of the patient will entail a healthy volunteer effect [73•]. The slow enrolment in conventional trials limits generalizability and validity of results [110] (Fig. 1). Conventional trials are further biased by experienced investigators and follow-up care that is more rigorous than routine care, leading to overestimation of benefit and underestimation of harm. From a patient and societal perspective, a broad view of any intervention, including magnitude of effect, real-world effectiveness, and cost-effectiveness, is important [73•].

A pragmatic or registry-based trial is embedded in routine clinical care. Although registry coverage is not complete and trial participation within a registry is selective, the population included in an RRCT will be more representative of the general HF population and findings more generalizable (Fig. 1). Mineralocorticoid receptor antagonists (MRAs) are highly effective in HFrEF trials, but there has been concern that in the real world, monitoring of renal function and electrolytes is not in adherence to guidelines [111], and observational studies suggest that MRAs may not be associated with benefit in the real world [112, 113]. MRAs remain class IA recommendation in HFrEF [5, 6] but these observations highlight the potential for differences in efficacy and effectiveness and indeed present the question of whether trials should adapt to real-world circumstances, or real-world care should adapt to trials.

### Costs of Developing and Testing a Novel Intervention vs. Testing a New Use Intervention

The cost of trials are added to the cost of drug development. Taking a single drug to market may cost $350 million, and given that 95% of drugs studied in humans fail to ultimately demonstrate safety and efficacy, the average cost may be higher than $5 billion [114]. The cost of inventing and developing a novel drug has been estimated to over $1 billion, and for 100 pharmaceutical companies reviewed by Forbes in 2013, the R&D costs per drug ranged from $15 million to $13 billion [114]. Industry R&D development is declining, and average peak-year sales of innovative products were forecasted to decline from $900 million in 2012 to $600 million in 2015 [115].

Much of this cost is related to the trial conduct itself. An estimated 18% of trial budgets are spent on supplementary or exploratory endpoints, and extraneous data collected in clinical trials cost drug developers $4 billion to $6 billion annually [116]. CV trials generally cost over $50 million [100] and mega trials in coronary disease, HF, and diabetes much more. A recent conventional trial of >14,000 diabetic patients enrolled at 660 sites cost nearly $250 million with monitoring constituting >$56 million (23%) [89••].

PCTs have been less expensive. The 7-year 42,000-patient ALLHAT cost $120 million to complete [67]. The ADAPTABLE trial that leverages EHR data to target enrollment of 20,000 patients over a shorter enrollment period is estimated to cost ≈$14 to 18 million with reduced costs for trial management and monitoring and increased costs for informatics [79•, 89••]. With access to both baseline and outcome data, RRCTs have unique possibilities to reduce cost further (Figs. 1 and 2). The incremental cost (beyond regular operations of the registry) in TASTE was $300,000, corresponding to about $50 per patient [41, 100].

### Costs of Using Novel vs. New Use Treatments

Given the need to recoup investment, the cost to patients and society of novel patented drugs brought to market are also high (Fig. 1). In HFrEF, the novel sacubitril/valsartan (Entresto®) costs $5 to >$10 per day in different Western countries. Despite convincing evidence from PARADIGM-HF, clear guidelines [5, 117], and emerging favorable cost-effectiveness data [118], reimbursement for sacubitril/valsartan remains variable. The high or low uptake of novel drugs such as sacubitril/valsartan over the next few years may serve as encouragement or deterrence, respectively, for industry to engage in new drug development for HF.

In contrast, HFpEF is uniquely positioned for testing of novel use of existing generic neurohormonal antagonist drugs. The cost of spironolactone in Sweden is less than 10 US cents per day and if proven effective in HFpEF, can have a tremendous impact for the many patients with HFpEF at low costs to society (Fig. 1). Similarly in AHF, trial-proven optimized use of the many generic and inexpensive drugs that are currently used empirically may deliver substantial benefit at low cost.

## Limitations of the RRCT and Future Challenges

As PCTs and RRCTs are encouraged by multiple stakeholders and becoming more familiar, it is important to recognize and address limitations, the importance of which are still difficult to assess. Will these trials be of high enough quality? How do we balance efficacy vs. effectiveness? As follow-up and monitoring is minimized for chronic interventions, how do we ensure the primary concern: safety to trial participants? How do we address privacy in these large patient databases, and will it be possible to reduce the need for informed consent? How important is, and what are additional costs of, blinding? Will pragmatic trials be able to assess effects on composite and patient-reported outcome measures (PROM), which are gaining in acceptance and importance, especially in chronic conditions associated with poor quality of life such as HFpEF? How can adjudication of these events be facilitated? Although a unique strength of PCTs and RRCTs is unselective inclusion and generalizable, relevant real-world findings, these trials have not yet been conducted in multinational or worldwide settings, limiting geographic generalizability. As we have developed the RRCT concept in Sweden, we also recognize that our population of 10 million is small, and it is in our interest to expand our RRCT methodology and RRCT platforms to other regions of the world.

### The Major Future Challenge: Funding

Although RRCTS are inexpensive, there remain fundamental components of GCP and trial infrastructure that entail considerable expense, generally beyond that possible from individual institutional or investigator grants. The medical products industry and public funders have previously not focused on pragmatic trials [67]. Industry has little incentive to fund RRCTs of generic drugs. However, it seems it would be attractive to collaborate with academia and registries in different forms of hybrid PCT or RRCT settings. Stakeholders including public regulatory, and funding agencies appear to recognize the need for trial reform [34, 37, 41, 67, 69•, 70, 71, 90, 92•, 94••, 96•, 101••] and should be willing to fund pragmatic trials. However, NIH extramural funding was an estimated 70% to basic and only 30% to clinical research of all types [119]. Now is the time for both industry and public funders to leverage the emerging PCT and RRCT infrastructure for efficiency and inexpensiveness, leading to new treatments for patients combined with savings for shareholders and the public.
